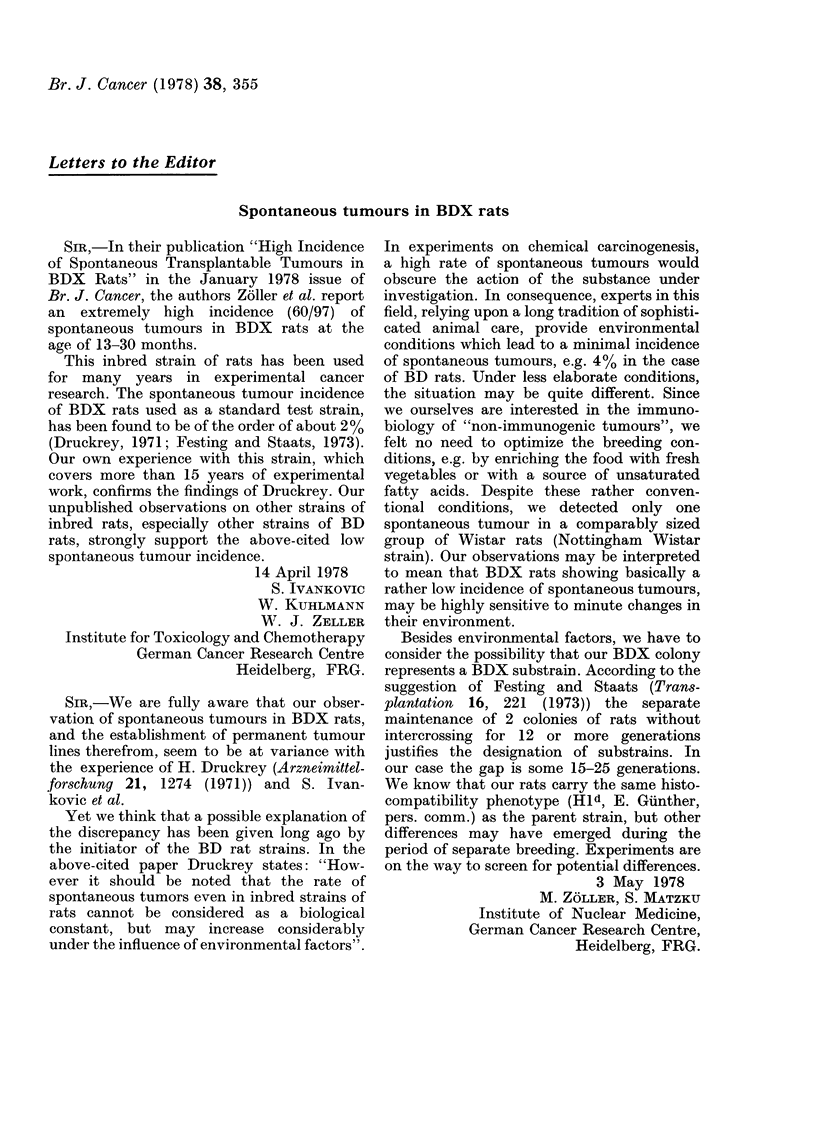# Spontaneous tumours in BDX rats.

**DOI:** 10.1038/bjc.1978.211

**Published:** 1978-08

**Authors:** S. Ivankovic, W. Kuhlmann, W. J. Zeller


*Br. J. Cancer* (1978) **38**, 355

### *Letters to the Editor*

### Spontaneous tumours in BDX rats

SIR,—In their publication "High Incidence of Spontaneous Transplantable Tumours in BDX Rats" in the January 1978 issue of *Br. J. Cancer*, the authors Zöller *et al.* report an extremely high incidence (60/97) of spontaneous tumours in BDX rats at the age of 13–30 months.

This inbred strain of rats has been used for many years in experimental cancer research. The spontaneous tumour incidence of BDX rats used as a standard test strain, has been found to be of the order of about 2% (Druckrey, 1971; Festing and Staats, 1973). Our own experience with this strain, which covers more than 15 years of experimental work, confirms the findings of Druckrey. Our unpublished observations on other strains of inbred rats, especially other strains of BD rats, strongly support the above-cited low spontaneous tumour incidence.

14 April 1978

S. IVANKOVIC
W. KUHLMANN
W. J. ZELLER
Institute for Toxicology and Chemotherapy
German Cancer Research Centre
Heidelberg, FRG.